# Fast subcellular localization by cascaded fusion of signal-based and homology-based methods

**DOI:** 10.1186/1477-5956-9-S1-S8

**Published:** 2011-10-14

**Authors:** Man-Wai Mak, Wei Wang, Sun-Yuan Kung

**Affiliations:** 1 Department of Electronic and Information Engineering, The Hong Kong Polytechnic University, Hong Kong; 2 Department of Electrical Engineering, Princeton University, USA

## Abstract

**Background:**

The functions of proteins are closely related to their subcellular locations. In the post-genomics era, the amount of gene and protein data grows exponentially, which necessitates the prediction of subcellular localization by computational means.

**Results:**

This paper proposes mitigating the computation burden of alignment-based approaches to subcellular localization prediction by a cascaded fusion of cleavage site prediction and profile alignment. Specifically, the informative segments of protein sequences are identified by a cleavage site predictor using the information in their N-terminal shorting signals. Then, the sequences are truncated at the cleavage site positions, and the shortened sequences are passed to PSI-BLAST for computing their profiles. Subcellular localization are subsequently predicted by a profile-to-profile alignment support-vector-machine (SVM) classifier. To further reduce the training and recognition time of the classifier, the SVM classifier is replaced by a new kernel method based on the perturbational discriminant analysis (PDA).

**Conclusions:**

Experimental results on a new dataset based on Swiss-Prot Release 57.5 show that the method can make use of the best property of signal- and homology-based approaches and can attain an accuracy comparable to that achieved by using full-length sequences. Analysis of profile-alignment score matrices suggest that both profile creation time and profile alignment time can be reduced without significant reduction in subcellular localization accuracy. It was found that PDA enjoys a short training time as compared to the conventional SVM. We advocate that the method will be important for biologists to conduct large-scale protein annotation or for bioinformaticians to perform preliminary investigations on new algorithms that involve pairwise alignments.

## Background

### Motivation of subcellular localization prediction

For a protein to function properly, it must be transported to the correct organelles of a cell and folded into correct 3-D structures. Therefore, knowing the subcellular localization of a protein is one step towards understanding its functions. However, the determination of subcellular localization by experimental means is often time-consuming and laborious. Given the large number of un-annotated sequences from genome projects, it is imperative to develop efficient and reliable computation techniques for annotating biological sequences.

In recent years, impressive progress has been made in the computational prediction of subcellular localization. A number of approaches have also been proposed in the literature. These methods can be generally divided into four categories, including predictions based on sorting signals [1-6], global sequence properties [7-10], homology [11-13] and other information in addition to sequences [14,15]. Methods based on sorting signals are very fast, but they typically suffer from low prediction accuracy. Homology-based methods are more accurate, but they are very slow. Therefore, *fast* and *reliable* predictions of subcellular localization still remain a challenge.

### Approaches to subcellular localization prediction

Signal-based methods predict the localization via the recognition of N-terminal sorting signals in amino acid sequences. PSORT, proposed by Nakai in 1991 [2], is one of the early predictors that use sorting signals for protein’s subcellular localization. PSORT and its extensions – WoLF PSORT [3,4] – derive features such as amino acid compositions and the presence of sequence motifs for localization prediction. In the late 90’s, researchers started to investigate the application of neural networks [16] to recognize the sorting signals. In a neural network, patterns are presented to the input layer of artificial neurons, with each neuron implementing a nonlinear function of the weighted sum of the inputs. Because amino acid sequences are of variable length, the input to the neural network is extracted from a short window sliding over the amino acid sequence. TargetP [17,18] is a well-known predictor that uses neural networks.

Another type of approaches relies on the fact that proteins of different organelles have different global properties such as amino-acid composition. Based on amino-acid composition and residue-pair frequencies, Nakashima and Nishikawa [10] developed a predictor that can discriminate between soluble intracellular and extracellular proteins. Another popular predictor based on amino acid composition is SubLoc [7]. In SubLoc, a query sequence is converted to 20-dim amino-acid composition vector for classification by support vector machines (SVMs). Recently, Xu et al. [19] proposed a semi-supervised learning technique (a kind of transductive learning) that makes use of unlabelled test data to boost the classification performance of SVMs. One limitation of composition-based methods is that information about the sequence order is not easy to represent. Some authors proposed using amino-acid pair compositions (dipeptide) [8, 9, 20] and pseudo amino-acid compositions [21] to enrich the representation power of the extracted vectors.

The homology-based methods use the query sequence to search protein databases for homologs [11,12] and predict the subcellular location of the query sequence as the one to which the homologs belong. This kind of method can achieve very high accuracy when homologs of experimentally verified sequences can be found in the database search [22]. A number of homology-based predictors have been proposed. For example, Proteome Analyst [23] uses the presence or absence of the tokens from certain fields of the homologous sequences in the Swiss-Prot database as a means to compute features for classification. In Kim et al. [24], an unknown protein sequence is aligned with every training sequences (with known subcellular locations) to create a feature vector for classification. Mak et al. [13] proposed a predictor called PairProSVM that uses profile alignment to detect weak similarity between protein sequences. Given a query sequence, a profile is obtained from PSI-BLAST search [25]. The profile is then aligned with every training profile to form a score vector for classification by SVMs.

Some predictors not only use amino acid sequences as input but also require extra information such as lexical context in database entries [14] or Gene Ontology entries [15] as input. Although studies have shown that this type of method can outperform sequence-based methods, the performance has only been measured on data sets where all sequences have the required additional information.

### Limitations of existing approaches

Among all the methods mentioned above, the signal-based and homology-based methods have attracted a great deal of attention, primarily because of their biological plausibility and robustness in predicting newly discovered sequences. Comparing these two approaches, the signal-based methods seem to be more direct, because they determine the localization from the sequence segments that contain the localization information. However, this type of method is typically limited to the prediction of a few subcellular locations only. For example, the popular TargetP [5,6] can only detect three localizations: chloroplast, mitochondria, and secretory pathway signal peptide. The homology-based methods, on the other hands, can in theory predict as many localizations as available in the training data. The downside, however, is that the whole sequence is used for the homology search or pairwise alignment, without considering the fact that some segments of the sequence are more important or contain more information than the others. Moreover, the computation requirement will be excessive for long sequences. The problem will become intractable for database annotation where tens of thousands of proteins are involved.

### Our proposal for addressing the limitations

Our earlier report [26] has demonstrated that computation time of subcellular localization based on profile alignment SVMs can be substantially reduced by aligning profiles up to the cleavage site positions of signal peptides, mitochondrial targeting peptides, and chloroplast transit peptides. Although 20-fold reduction in total computation time (including alignment, training and recognition time) has been achieved, the method fails to reduce the profile creation time, which will become a substantial part of the total computation time when the database becomes large. In this paper, we propose a new approach that can reduce both the profile creation time and profile alignment time. In the new approach, instead of cutting the profiles, we shorten the sequences by cutting them at the cleavage site locations. The shortened sequences are then presented to PSI-BLAST to compute the profiles. To further reduce the training and recognition time of the classifier, we propose replacing the SVMs by kernel perturbation discriminants.

### Fusion of signal- and homology-based methods

**Figure 1:**
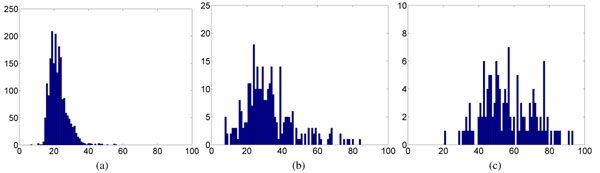
**Length distribution of SP, mTP, and cTP.** The histograms of length of (a) secretory pathway signal peptides, (b) mitochondrial targeting peptides, and (c) chloroplast transit peptides. The length is the number of amino acids from the N-terminus up to the cleavage site. *Vertical axes*: number of occurrences. *Horizontal axes*: sequence length.

Fig. 1 shows the histograms of the length of signal pep-tides (SP), mitochondrial transit peptides (mTP), and chloroplast transit peptides (cTP). The length is the number of amino acids from the N-terminus up to the cleavage site. It is obvious that the lengths of these pep-tides are rather short. Given the fact that the majority of proteins in the Swiss-Prot database have about a few hundred amino acids and that some proteins could have length longer than 5,000 amino acids, tremendous computational saving can be achieved by combining the signal-based and homology-based methods described below.

### Truncation of profiles/sequences

**Figure 2:**
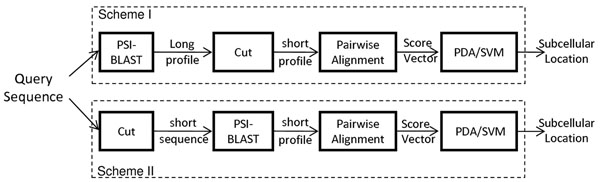
**Two schemes for computation saving.** Two schemes for reducing the computation of the subcellular localization process. In Scheme I, a full-length query sequence is presented to PSI-BLAST for computing a full-length profile; then the profile is truncated at the predicted cleavage site. The truncated profile is then aligned with all of the truncated training profiles to produce a profile-alignment score vector for classification. In Scheme II, the query sequence is truncated at the predicted cleavage site before inputting to PSI-BLAST for computing the profile. The cleavage sites are predicted by CSitePred [27] or TargetP [5].

We have investigated two fusion schemes (see Fig. 2):

I: *Truncating Profiles*. Given a query sequence, we pass it to PSI-BLAST [25] to determine a full-length profile (PSSM and PSFM [13]). The profile is then truncated at the cleavage site position. The truncated profile is aligned with each of the training profiles to create a vector for classification. Note that the training profiles are also created by the same procedure.

II: *Truncating Sequences*. Given a query sequence, we truncate it at the cleavage site and pass the truncated sequence to PSI-BLAST to determine a short-length profile. The profile is then aligned with all of the training profiles to create a vector for classification. All training profiles are also created by the same procedure.

Note that as the time taken by PSI-BLAST search (profile-creation time) is proportional to the query sequence, Scheme II is expected to provide more computation saving than Scheme I. However, as the sequences are truncated at an early stage, important information may be lost if cleavage site prediction is inaccurate. The “Results and Discussion” Section provides experimental evidences suggesting that Scheme II can provide significant computation saving without suffering from severe information loss.

### Cleavage site prediction

**Figure 3:**
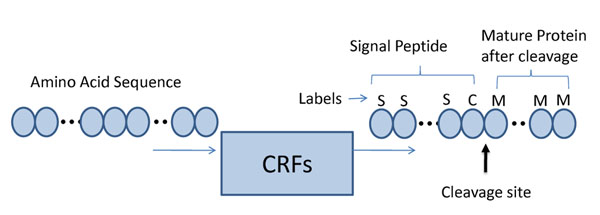
**CRFs for cleavage site prediction.** Conditional random fields (CRFs) for cleavage cite prediction. Given a sequence of observations, each amino acid in the sequences is labelled as either “Signal”, “Cleavage”, or “Mature”, e.g., SSSSSSCMMMMMM. The cleavage site is located at the transition between C and M.

**Figure 4:**
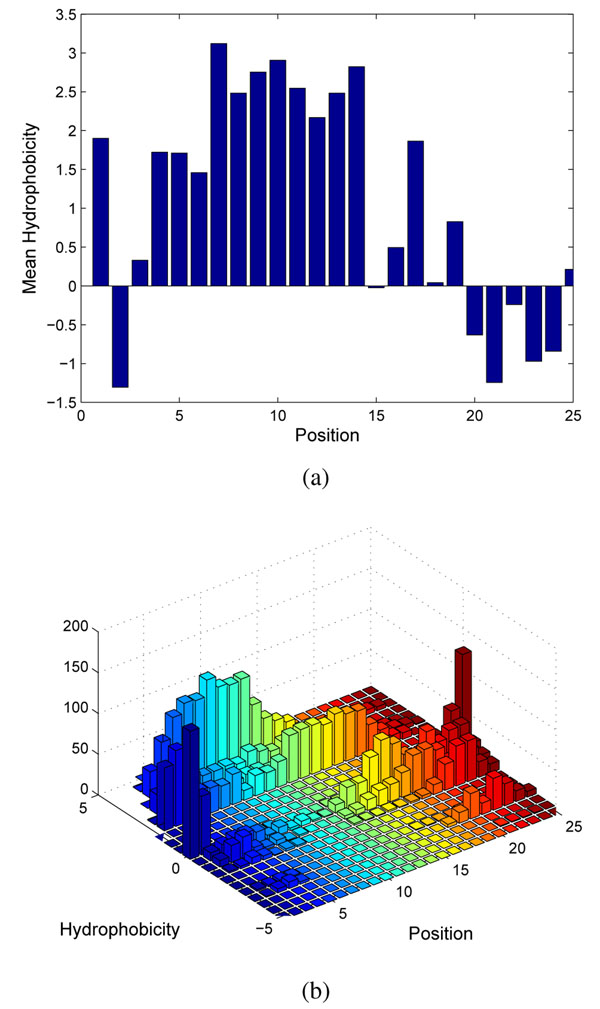
The mean (top) and the histograms (bottom) of hydrophobicity of 179 signal peptides at different sequence positions. The cleavage site of these sequences is between Positions 19 and 20.

This work investigated two cleavage site predictors: conditional random fields (CRFs) [27,28] and TargetP [5,6]. CRFs [29] were originally designed for sequence labelling tasks such as Part-of-Speech (POS) tagging. Given a sequence of observations, a CRF finds the most likely label for each of the observations. To use CRFs for cleavage site prediction, amino acid sequences are treated as observations and each amino acid in the sequences is labelled as either Signal, Cleavage, or Mature, e.g., SSSSSSCMMMMMM, as illustrated in Fig. 3. The cleavage site is located at the transition between C and M. Amino acids of similar properties can be categorized according to their hydrophobicity and charge/polarity as shown in Table 1. These properties are used because the h-region of signal peptides is rich in hydrophobic residues and the c-region is dominated by small, non-polar residues [30]. Moreover, as illustrated in Fig. 4, the degree of hydrophobicity is also very different at different positions, making this feature useful for the labelling task.

**Table 1:** Grouping of amino acids according to their hydrophobicity and charge/polarity [43].

Property	Group
Hydrophobicity	H1={D,E,N,Q,R,K}
	H2={C,S,T,P,G,H,Y}
	H3={A,M,I,L,V,F,W}

Charge/Polarity	C1={R,K,H}
	C2={D,E}
	C3={C,T,S,G,N,Q,Y}
	C4={A,P,M,L,I,V,F,W}

TargetP is one of the most popular signal-based sub-cellular localization predictors and cleavage site predictors. Given a query sequence, TargetP can determine its subcellular localization and will also invoke SignalP [31], ChloroP [32], or a program specialized for mTP to determine the cleavage site of the sequence. TargetP requires the N-terminal sequence of a protein as input. During prediction, a sliding window scans over a query sequence; for each segment within the window, a numerically encoded vector is presented to a neural network to compute the segment score. The cleavage site is determined by finding the position at which the score is maximum. The cleavage site prediction accuracy of SignalP on Eukaryotic proteins is around 70% [33] and that of ChloroP on cTP is 60% (±2 residues) [32].

## Methods

### Data preparation

Protein sequences with experimentally annotated subcellular locations were extracted from the Swiss-Prot Release 57.5 according to the following criteria.

1. Only the entries of Eukaryotic species, which were annotated with “Eukaryota” in the OC (Organism Classification) fields in Swiss-Prot, were included.

2. Entries annotated with ambiguous words, such as “probable”, “by similarity” and “potential”, were excluded because of the lack of experimental evidence.

3. Sequences annotated with “fragment” were excluded.

4. For signal peptides, mitochondria, and chloroplast, only sequences with experimentally annotated cleavage sites were included.

The extracted sequences were then filtered by BLAST-Clust [34] so that the resulting sequences have sequence identity less than 25%. Table 2 shows the breakdown of the dataset. A modified version of the Perl scripts provided by [35] was used for creating the dataset.

**Table 2:** Breakdown of eukaryotic dataset derived from the Swiss-Prot database (release 57.5).

Class Index	Subcellular Location	Number of Proteins
1	Extracellular	693
2	Mitochondria	167
3	Chloroplast	74
4	Others(Cytoplasm/Nucleus)	1617

		2552(total)

### PDA and SVM for multi-class classification

We used perturbational discriminant analysis (PDA) [36] and support vector machines (SVMs) [37] for classification. The formulation of PDA can be found in the Appendix. During the training phase, *N* training profiles were obtained by Scheme I or Scheme II. Pair-wise profile-alignments were then performed to create an *N* × *N* symmetric score matrix ***K***, which were then used to train the PDA and SVM classifiers as follows.

#### One-vs-rest PDA and SVM classifier

A *C*-class problem can be formulated as *C* binary classification problems in which each problem is solved by a binary classifier. Given the training sequences of *C* classes, we trained *C* PDA score functions:(1)

where ***x*** is a query sequence,  contains the similarity (via profile alignment) between ***x*** and the *N* training profiles, and ***a****_i_* and *b_i_* were obtained by Eq. 11 and Eq. 12 in the Appendix.

For the SVM classifier, the score functions in Eq. 1 are replaced by the linear SVM score functions:

where *a_ij_*’s are the Lagrange multipliers of Class *i*, and *y_ij_* = 1 if ***x****_j_* belongs to Class *i* and *y_ij_* = –1 otherwise. Then, given a test sequence ***x***, the class label is given by

#### Cascaded fusion of PDA and SVM

Instead of using Eqs. 11 and 12, the optimal weights in PDA can also be equivalently expressed in terms of ***d*** and *η* in Eqs. 8 and 9. In a *C*-class problem, the *i*-th class will have its corresponding ***d****_i_* and *η_i_*, where *i* = 1,*…*,*C.* However, because of the dependence in ***d****_i_*, the rank of matrix [***d***_1_, …, ***d****_C_*] is *C –* 1. Therefore, there are *C –* 1 independent sets of PDA parameters:

where **1** is an *N-*dim vector of all 1’s and *p* is a perturbation parameter. During recognition, an unknown sample *x* is projected onto a (*C –* 1)-dim PDA space spanned by [***a***_1_,*…*,***a***_C--1_] using

***g***(***x***) = ***Â*^T^*k***(***x***) + [*b*_1_,*…*, *b_C–_*_1_]^T^, *g*(***x***) ∊ ℜ*^C^*^–1^*.*

**Figure 5:**
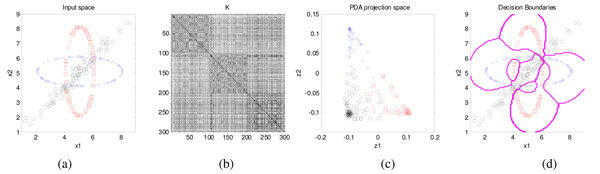
Multi-class classification by perturbational discriminant analysis (PDA). (a) 2-dim data {*x*_1_,…,*x_N_*} of 3 different classes in the input space, where *N* = 300*.* (b) *N* x *N* RBF kernel matrix *K*, where *k_i_*_,_*_j_* = exp{*-||****x****_i_ - x_j_||*^2^/2}*;* each column of ***K*** is an *N*-dim vector ***k***(***x****_i_*) in the empirical space *K.* (c) Projected data *g*(***x****_i_*) = *Â*^T^***k***(***x_i_***) + ***b*** on the PDA space where data can be easily classified by 1-vs-rest SVMs. (d) Decision boundaries produced by PDAproj+SVM.

Then, ***g***(***x***) is classified by one-vs-rest RBF-SVMs. In the sequel, we refer to this cascaded fusion as PDAproj+SVM. Fig. 5 exemplifies the capability of PDAproj+SVM using a 2-dim multi-class problem.

### Performance evaluation

We used 5-fold cross validation to evaluate the performance. The overall prediction accuracy, the accuracy for each subcellular location, and the Matthew’s correlation coefficient (MCC) [38] were used to quantify the prediction performance. MCC allows us to overcome the shortcoming of accuracy on unbalanced data [38].

We measured the computation time on a Core 2 Duo 3.16GHz CPU running Matlab and SVMlight. The computation time was divided into profile creation time, alignment time, classifier training time, and classification time.

## Results and discussion

### Performance of cleavage site prediction

Table 3 shows the cleavage site prediction accuracy of TargetP and CSitePred [28] (a CRF-based predictor). It suggests that CSitePred is better than TargetP(P) in terms of predicting the cleavage sites of signal peptide (SP) but is poorer than TargetP(N). The results also suggest that while CSitePred is slightly inferior to TargetP in predicting the cleavage sites of mitochondria, it is significantly better than TargetP in predicting the cleavage sites of chloroplasts. Note that the overall accuracies depend heavily on the SP class because of the large number of signal peptides in the dataset (see Table 2).

**Table 3:** Cleavage-site prediction accuracies achieved by TargetP and CSitePred. For TargetP, (P) and (N) mean using the ‘Plant’ and ‘Non-plant’ option of the predictor, respectively. TargetP will invoke SignalP, ChloroP, or a program specialized in predicting mTP for cleavage site prediction. CSitePred is based on conditional random fields.

Cleavage Site Predictor	Cleavage Site Prediction Accuracy (%)			
	
	SP	mTP	cTP	Overall
TargetP(P)	71.49	44.04	8.82	64.55
TargetP(N)	84.63	46.69	2.21	75.28
CSitePred	79.40	39.40	31.62	71.73

The prediction accuracy of chloroplasts by TargetP shown in Table 3 is significantly lower than that in [32]. There are two reasons for this difference: (1) our dataset has sequence identity lower than that of [32] and (2) we consider predicting precisely the ground-truth sites as correct predictions whereas [32] considers predictions within ±2 positions of the ground-truth sites as correct predictions. In fact, if we relaxed the criterion of correct prediction to ±2 ground-truth positions, the prediction accuracy on chloroplasts achieved by TargetP increases to 47.06%.

### Sensitivity analysis

To evaluate the effect of incorrect cleavage site prediction on the accuracy of subcellular localization, sensitivity analysis was performed by truncating SP, mTP, and cTP at the ground-truth cleavage sites and plus/minus several positions of the ground-truths. Specifically, the sequence cut-off positions are 16, 8, and 2 amino acids upstream and 2, 16, 32, and 64 amino acids downstream from the ground-truth cleavage site.

**Figure 6:**
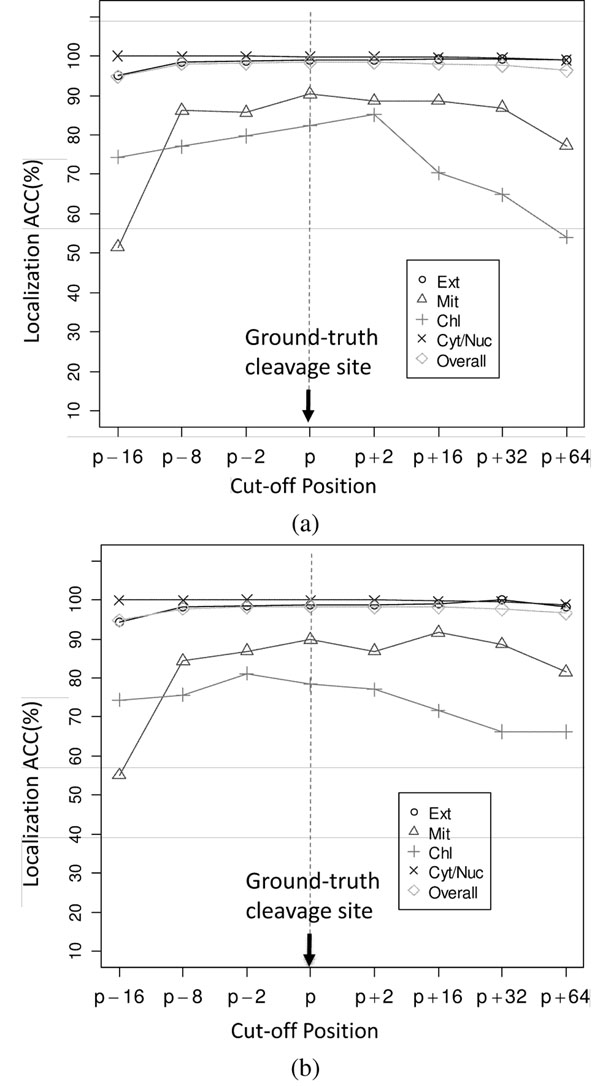
Sensitivity of subcellular localization accuracy with respect to the (top) profile cut-off positions in Scheme I and (bottom) sequence cut-off positions in Scheme II (see Fig. 2). *p* is the ground-truth cleavage site. For “Cyt/Nuc” proteins, *p* is set to 170.

Fig. 6 shows that the overall accuracy of subcellular localization does not rely significantly on the precision of cleavage site prediction as long as the predicted sites are not too far away from the ground-truths.

Apparently, mTP and cTP are more sensitive to the error of cleavage site prediction, which agrees with the fact that the signals of mTP and cTP are weaker. Localization performance of these sequences degrades when the cut-off position drifts away significantly the ground-truth cleavage site. But the overall accuracy can be maintained at above 95% even if the drift is as large as –16 and +64 positions from the ground-truth. Moreover, a forward drift of 64 positions from the ground truth cleavage site leads to a higher overall accuracy when compared to that of a backward drift of 16 positions, which suggests that cutting sequences before their cleavage sites may lose useful information in the signal pep-tides while including extra (may be irrelevant) information by cutting sequences after their cleavage sites is not detrimental to subcellular location accuracy.

### Profile-creation time

**Figure 7:**
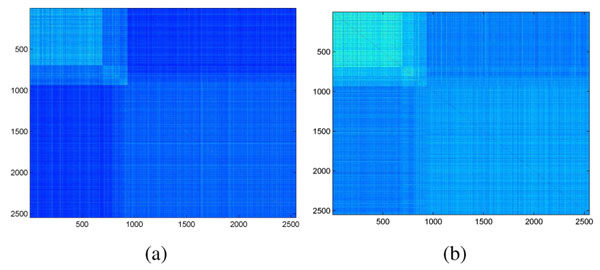
Profile-alignment score matrices produced by (left) Scheme I and (right) Scheme II in Fig. 2.

Fig. 7 shows the score matrices obtained by the two profile creation schemes illustrated in Fig. 2. The figure shows that the two alignment score matrices exhibit a similar pattern, suggesting that classifiers based on these matrices will produce similar classification accuracy. This argument is confirmed by Table 4, which shows that cutting the sequences at cleavage sites before inputting to PSI-BLAST can reduce the profile creation time by 6 times without significant reduction in subcellular localization accuracy.

**Table 4:** Average computation time to create a profile by PSI-BLAST using sequences of different length as input. In Scheme I, full-length sequences are presented to PSI-BLAST and the resulting profiles are truncated at the predicted cleavage sites. In Scheme II, truncation is applied to the sequences before presenting to PSI-BLAST. In both cases, CRFs (CSitePred) were used to predict the cleavage sites.

Scheme	Input to PSI-BLAST	Profile Creation Time (second)	Subcellular Localization Accuracy
I	Full-length sequences	30.5	91.69%
II	Sequences truncated at predicted cleavage sites	4.7	91.45%

### Profile-alignment time

Table 5 shows that the computation time for full-length profile alignment is striking — nearly thirty-five seconds per sequence, which suggests that full-length alignment is computationally prohibitive. Therefore, it is imperative to limit the length of the sequences or profiles before alignment. Table 5 also shows that truncating the sequences at their cleavage site positions leads to nearly a 20 folds reduction in alignment time without suffering from loss in subcellular localization performance. This is because the signal segment can be found in the N-terminus, and removing the amino acids beyond the cleavage site helps the alignment focuses on the relevant features in the profiles and disregard noise.

**Table 5:** Profile-Alignment time and subcellular localization accuracy for different sequence cut-off positions in Scheme II. In the first column, “Full length” means that no sequence truncation was applied. “TargetP(P)” and “Tar-getP(N)” mean that the cutoff position is determined by TargetP using the “Plant” option and “Non-plant” option, respectively. CSitePred is a cleavage site predictor based on conditional random fields.

Seq. Cutoff position	Alignment Time for Each Profile (sec.)	Subcellular Localization Accuracy (%)
Full length	34.7	91.64
170	4.7	90.98
Ground-truth	1.9	98.31
Determined by TargetP(P)	1.8	89.08
Determined by TargetP(N)	1.7	93.14
Determined by CSitePred	1.9	91.45

### SVM versus PDA

Table 6 shows that the training time of PDA and PDAproj+SVM are only one-fifth of that of SVM. However, the accuracy of PDA and PDAproj+SVM are lower than that of SVM.

**Table 6:** The computation time and performance of different classifiers in the subcellular localization task. The classification time is the time to classify a profile-alignment score vector with dimension equal to the number of training vectors. The training time is the time required to train a classifier, given a profile-alignment score matrix *K*. In PDAproj+SVM, PDA was applied to project the samples in the input space to a (*C* - 1)-dim space (*C* = 4 here); the projected vectors were then classified by RBF-SVMs.

Classification Method	Training Time (sec.)	Classification Time (sec.)	SubLoc Acc.
SVM	51.4	0.7	91.45%
PDA	9.9	1.9	90.24%
PDAproj+SVM	8.9	0.1	89.97%

### Compared with state-of-the-art predictors

We compared the accuracy of the proposed fusion of signal-based and homology-based methods with SubLoc [7], TargetP [5] and PairProSVM [13]. Table 7 shows that the overall accuracy of the proposed method (the 5th row) is 5.2% higher than that of TargetP (3rd row) and is significantly better than that of SubLoc (1st row). Our method outperforms TargetP in Ext (SP) and Cyt/Nuc prediction while performing worse than TargetP in predicting Mit and Chl. One limitation of TargetP is that users need to select either “Plant” or “Non-plant”. If the former is selected, the performance of Ext and Cyt/Nuc degrade significantly, leading to a low overall accuracy; if the latter is selected, none of the chloroplast proteins can be correctly predicted. The cascaded fusion of cleavage site prediction and PairProSVM, on the other hand, can classify all four classes with fairly high accuracy, leading to a higher overall accuracy.

**Table 7:** Subcellular localization performance achieved by different classifiers. The second column specifies the cleavage site predictors that were used for determining the positions at which the amino sequences were truncated. Notice that TargetP can perform both cleavage site prediction and subcellular localization. For Rows 4 and 5, TargetP was used as a cleavage site predictor, where “TargetP(P)” and “TargetP(N)” mean selecting plant or non-plant option in TargetP, respectively. For Rows 6–8 “CRF” means that conditional random fields were used for cleavage site prediction.

Row	Cleavage Site Predictor	Localization Predictor	Classification Accuracy (%)					Matthew’s correlation coefficient (MCC)				
			
			Ext	Mit	Chl	Cyt/Nuc	Overall	Ext	Mit	Chl	Cyt/Nuc	Overall
1	—	SubLoc [7]	51.44	55.83	—	77.86	66.79	—	—	—	—	—
2	—	TargetP (P)	79.08	88.02	89.19	69.57	73.93	0.79	0.49	0.79	0.64	0.65
3	—	TargetP (N)	97.40	89.22	0.00	87.82	87.97	0.93	0.58	0.00	0.81	0.84

4	TargetP(N)	SVM	97.26	67.07	36.49	95.86	92.63	0.93	0.70	0.53	0.86	0.90
5	TargetP(N)	PDA	97.55	61.68	6.76	95.61	91.34	0.91	0.68	0.26	0.84	0.88
6	TargetP(N)	PDAproj+SVM	97.26	65.27	37.84	93.57	91.10	0.93	0.64	0.50	0.83	0.88
7	CRF	SVM	94.52	63.47	28.38	95.86	91.45	0.90	0.68	0.45	0.84	0.89
8	CRF	PDA	94.81	59.28	1.35	95.55	90.24	0.88	0.67	0.11	0.82	0.81
9	CRF	PDAproj+SVM	94.66	63.47	25.68	93.63	89.97	0.90	0.60	0.41	0.82	0.87

The prediction accuracy and MCC of the proposed methods (Rows 4–10 in Table 7) are comparable to Pair-ProSVM (Row 4 in Table 7). The main improvement is on computation time reduction.

Because ChloroP is weak in predicting the cleavage sites of chloroplasts (see Table 3), it is not a good candidate for assisting PairProSVM. This is evident by the low subcellular localization accuracy of chloroplasts in Table 7 when TargetP is used as a cleavage site predictor. However, TargetP is fairly good at predicting the subcellular location of chloroplasts when it is used as a localization predictor.

Among the four classes in Table 7, the subcellular localization accuracies of mitochondria and chloroplasts are generally lower than that of Ext and Cyt/Nuc. The reason may be that these transit peptides are less well characterized and their motifs are less conserved than those of secretary signal peptides [6].

Table 7 also suggests that the TargetP(N) is very effective in assisting PairProSVM, leading to the highest prediction accuracy (92.6%) among all subcellular localization predictors. In particular, except for predicting Chl, TargetP in combination with PairProSVM can surpass the other methods in subcellular localization accuracy and MCC.

## Conclusions

This paper has demonstrated that homology-based sub-cellular localization can be speeded up by reducing the length of the query amino acid sequences. Because shortening an amino acid sequence will inevitably throw away some information in the sequence, it is imperative to determine the best truncation positions. This paper shows that these positions can be determined by cleavage site predictors such as TargetP and CSitePred. The paper also shows that as far as localization accuracy is concerned, it does not matter whether we truncate the sequences or truncate the profiles. However, truncating the sequence has computation advantage because this strategy can save the profile creation time by as much as 6 folds.

## Appendix: kernel discriminant analysis

This appendix derives the formulations of kernel discriminant analysis. The key idea lies in the equivalency between the optimal projection vectors in the Hilbert space, spectral space and empirical space.

### Input, Hilbert, spectral, and empirical Spaces

Denote the mapping from an input space *X* into a Hilbert space *H* as:

In bioinformatics, *X* is a vectorial space for microarray data and a sequence space for DNA or protein sequences. Given a training dataset {***x***_1_,…, ***x****_N_*} in *X* and a kernel function *K*(***x***, ***y***), an object can be represented by a vector of similarity with respect to all of the training objects [39]:

This *N*-dim space, denoted by *K*, is called empirical space. The associate kernel matrix is defined as

The construction of the empirical space for vectorial and non-vectorial data are quite different. For the former, the elements of ***K*** are a simple function of the corresponding pair of vectors in *X*. For the latter, the elements in ***K*** are similarities between the corresponding pairs of objects.

The kernel matrix ***K*** can be factorized with respect to the basis functions in *H:****K*** = **Φ**^T^**Φ**, where . Alternatively, it can be factorized via spectral decomposition:  where .

Denote the *i*-th row of ***E*** as ***e*^(^*^i^*^)^** [e^(^*^i^*^)^(***x***_1_),…,e^(^*^i^*^)^(***x****_N_*)]. Because  the rows of ***E*** exhibit a vital orthogonality property:

where λ*_i_* is the *i*-th element of the diagonal of **Λ**.

For any positive-definite kernel function *K*(***x***, ***y***) and training dataset {***x***_1_,…,***x_N_***} in ***X***, there exists a (nonlinear) mapping from the original input space ***X*** to an *N*-dim spectral space *E*:

Note that ***K*** = ***E***^T^***E***, i.e., . Therefore, .

Many kernel-based machine learning problems involve finding optimal projection vectors in *H*, *E*, and *K*, which will be respectively denoted as ***w***, ***v***, and ***a****.* It can be shown [36] that the projection vectors are linearly related as follows:(2)

where we have used the relationships ***w*** = **Φ*a*** and ***v*** = ***Ea****.*

### Orthogonal hyperplane principle (OHP)

Assume that the dimension of *H* is *M* and that the training data in *H* are mass-centered. When *M* >***N***, all of the ***N*** training vectors  will fall on an **(***M* –1)-dim *data hyperplane.* Mathematically, the data-hyperplane is represented by its normal vector ***p*** such that **Φ^T^*p*** = **1**. The optimal decision-hyperplane in *H* (represented by ***w***) must be orthogonal to the data-hyperplane:

***w***^T^***p*** = 0 ⇒ **α**^T^**Φ**^T^***p*** = 0 ⇒ **α**^T^1 = 0*.*

### Kernel Fisher discriminant analysis (KFDA)

The objective of KFDA [40] is to determine an optimal discriminant function (linearly) expressed in the Hilbert space ***H****:*

where *b* is a bias to account for the fact that training data may not be mass-centered. The discriminant function may be equivalently expressed in the *N*-dim spectral space *E:*

The finite-dimensional space *E* facilitates our analysis and design of optimal classifiers. In fact, the optimal projection vector ***v***_opt_ in *E* can be obtained by applying conventional FDA to the column vectors . To derive the objective function of KFDA, let us define(3)

where  and **1**_+_ and **1_â€“_** contain 1’s inentries corresponding to Classes *C***_+_** and *C_â€“_*, respectively, and 0’s otherwise; and ***N*_+_** and ***N****_-_* are the number of training samples in classes ***C*_+_** and ***C****_-_*, respectively. It can be shown that the objective function of KFDA is:(4)

where **1** is an *N-*dim vector with all elements equal to 1 and  and  are between-class and within-class covariance matrices in *E* space, respectively.

### Perturbational discriminant analysis (PDA)

The FDA and KFDA are based on the assumption that the observed data are perfectly measured. It is however crucial to take into account the inevitable perturbation of training data. For the purpose of designing practical classifiers, we can adopt the following perturbational discriminant analysis (PDA).

It is assumed that the observed data is contaminated by additive white noise in the spectral space. Denote the center-adjusted matrix of ***E*** as ***Ē*** and the uncorrelated noise as ***N***, then the perturbed scattered matrix is

where *ρ* is a parameter representing the noise level. Its value can sometimes be empirically estimated if the domain knowledge is well established a priori. Under the perturbation analysis, the kernel Fisher score in Eq. 4 is modified to the following perturbed variant:(5)

By taking the derivative of *J*_PDA_(***V***) with respect to ***V***, the optimal solution to Eq. 5 can be obtained as:

and using the Sherman-Morrison-Woodbury identity it can be shown that [41](6)

where *η* is a scalar whose value can be determined through the optimal solution in *K* space as follows.

Recall from Eq. 2 that dot-products in the three spaces are equivalent. Therefore, the discriminant function in *K* space can be written as:(7)

Given the optimal solution *v*_opt_ in the *E* space, the corresponding optimal solution in the *K* space is^1^(8)

where we have used ***K*** = ***U*^T^Λ*U*** and . Note that unlike Eq. 6, Eq. 8 does not require spectral decomposition, thus offering a fast close-form solution. Now using the orthogonal hyperplanes principle, we have(9)

Note that unlike Eq. 6, Eq. 8 does not require spectral decomposition, thus offering a fast close-form solution. Also, Eq. 6 suggests that *ρ* has more regularization effect on the minor components with small eigenvalues than on the major components with large eigenvalues. This serves well the purpose of regularization. Consequently, a PDA classifier will use less proportion of minor (and risky) components and more of major components. Therefore, the parameter *p* plays two major roles: (1) it can assure the Mercer condition and invertibility of the kernel matrix; and (2) it can suppress the weights assigned to the risker and less resilient components.

The remaining unknown is the bias *b.* Recall from Eq. 2 that dot-products in the three spaces are equivalent. Therefore, the discriminant function in *K* space can be written as:(10)

Putting all training data ***x****_i_* into Eq. 10, we have

where *y_i_* = 1 when ***x****_i_* ∊ *C*_+_ and *y_i_* = –1 when *x_i_* ∊ *C*_–_. Since ***K*** is invertible, we have *a*_opt_ = ***K***^–1^(***y***–*b***1**)*.* Eqs. 6 and 8 suggest that perturbation in the spectral space can be represented by shifting the diagonal of ***K*** by *p.* Therefore, taking the perturbation in the spectral space into account, we have

*a*_opt_ = (***K*** + *ρ****I***)^–1^ (***y****–b***1**)*.* (11)

Note that the solutions given in Eq. 8 and Eq. 11 are equivalent. Now, *b* can be determined by using the orthogonal hyperplane principle to maximize the inter-class separability:(12)

Note that the solutions of ***a*** and *b* in Eqs. 11 and 12 are equivalent to the least-squares SVM [42], although the way to derive the solutions are different.

## Authors' contributions

M.W. Mak and W. Wang contribute to (1) the idea of cascaded fusion of signal-based and homology-based methods, (2) preparation of data, (3) implementation of CRF cleavage site predictor and SVM/PDA classifiers, and (4) experimental evaluations. S.Y. Kung contributes to (1) the theoretical development and derivation of PDA and (2) the idea of cascaded fusion and sensitivity analysis.

## Competing interests

The authors declare that they have no competing interests.
